# Biologic and small molecule therapies for psoriasis in individuals with Down syndrome: Two cases and a systematic review

**DOI:** 10.1177/2050313X251359029

**Published:** 2025-07-22

**Authors:** Michal Moshkovich, Jonah W. Perlmutter, Ronald Vender

**Affiliations:** 1 Temerty Faculty of Medicine, University of Toronto, Toronto, ON, Canada; 2 Faculty of Health Sciences, McMaster University, Hamilton, ON, Canada

**Keywords:** psoriasis, small molecules, biologics, down syndrome, trisomy 21

## Abstract

Down syndrome (DS), also known as trisomy 21, is a genetic condition linked to a higher prevalence of skin disorders, including psoriasis, which affects up to 8% of individuals. DS patients with psoriasis present unique management considerations, including a theoretical increased risk of infectious complications with immunosuppressive therapies. This report includes two cases and a systematic review summarizing available evidence on psoriasis characteristics and treatment outcomes in individuals with DS. We report two DS patients with psoriasis demonstrating variable therapeutic responses: one controlled with acitretin and another requiring secukinumab after multiple treatment failures. To contextualize these findings, we conducted a systematic review following PRISMA guidelines, identifying 10 studies comprising 37 DS patients with psoriasis. Methotrexate was the most frequently failed therapy. Biologics targeting IL-17 and IL-23 pathways achieved the highest rates of complete resolution. These findings reflect Th1/Th17-driven inflammation in DS and highlight the need for individualized, pathway-specific management strategies.

## Case report

Herein, we present two cases of psoriasis in individuals with DS, illustrating variability in treatment response.^1^ The first is a 37-year-old East Indian male with a 5-year history of psoriasis involving 6% body surface area (BSA) with a psoriasis area severity index (PASI) score of 6 and scalp involvement. Despite failing topicals, he has maintained disease control on acitretin (10 mg daily) for 5 years. The second case is a 40-year-old Caucasian female with psoriasis for 25 years, previously affecting 12% BSA with a PASI of 13.2 and concurrent psoriatic arthritis, both now well controlled. She failed multiple agents including topicals, acitretin, methotrexate, and golimumab, before achieving long-term control on secukinumab (300 mg monthly) for 7 years. These cases highlight treatment response heterogeneity in DS patients, reinforcing the need for individualized therapeutic approaches.

## Discussion

The variability observed in biologic efficacy among DS patients is further reflected in the literature. Following PRISMA guidelines, MEDLINE and Embase databases were searched with keywords (Supplemental Table 1). Ten studies comprising 37 patients were included (Supplemental Table 2, Supplemental Table 3). The mean age was 23.3 years (range: 6–48) with 64.9% (24/37) males. Joint involvement occurred in 35.1% (13/37) and comorbidities in 59.5% (22/37) of patients with obesity most prevalent (29.7%, 11/37). The mean psoriasis duration was 103.5 months, with mean pretreatment PASI and BSA of 23.6% and 26.1%, respectively. Methotrexate was the most frequently failed therapy (39.5%, 17/43), aligning with our second case.

There were 63 documented biologic and small molecule therapies, including tumor necrosis factor (TNF)-α inhibitors (47.6%, 30/63), interleukin (IL)-12/23 inhibitors (20.6%, 13/63), and IL-17 Inhibitors (14.3%, 9/63), Janus Kinase (JAK) inhibitors (6.3%, 4/63), IL-23 inhibitors (7.9%, 5/63), and IL-6 inhibitors (3.2%, 2/63). The most common agents were ustekinumab (20.6%, 13/63), adalimumab (27%, 17/63), and etanercept (12.7%, 8/63). Treatment duration was reported in 22.2% (14/63) of cases, with a mean of 49 weeks (range: 6-208). Complete resolution occurred most frequently with tofacitinib (100%, 4/4), risankizumab (100%, 2/2), ustekinumab (69.2%, 9/13), ixekizumab (66.7%, 2/3), and guselkumab (66.7%, 2/3) (Supplemental Table 4), suggesting promising responses to IL-17 and IL-23 inhibitors. The sustained disease control in our second case supports the effectiveness of IL-17 blockade.

DS patients exhibit elevated interferon-gamma levels and type 1 T-helper (Th1) cells, creating a proinflammatory milieu that sustains a positive feedback loop, perpetuating psoriasis. TNF-α and IL-17, central mediators of the Th1 and Th17 pathways, further drive this dysregulation.^2^ Study limitations include potential reporting bias and a small sample size. Larger-scale studies are needed to refine therapeutic strategies involving TNF- α and IL-17/23 inhibitors tailored to DS immunopathophysiology.

## Supplemental Material

sj-docx-1-sco-10.1177_2050313X251359029 – Supplemental material for Biologic and small molecule therapies for psoriasis in individuals with Down syndrome: Two cases and a systematic review Supplemental material, sj-docx-1-sco-10.1177_2050313X251359029 for Biologic and small molecule therapies for psoriasis in individuals with Down syndrome: Two cases and a systematic review by Michal Moshkovich, Jonah W. Perlmutter and Ronald Vender in SAGE Open Medical Case Reports

sj-docx-2-sco-10.1177_2050313X251359029 – Supplemental material for Biologic and small molecule therapies for psoriasis in individuals with Down syndrome: Two cases and a systematic review Supplemental material, sj-docx-2-sco-10.1177_2050313X251359029 for Biologic and small molecule therapies for psoriasis in individuals with Down syndrome: Two cases and a systematic review by Michal Moshkovich, Jonah W. Perlmutter and Ronald Vender in SAGE Open Medical Case Reports

sj-docx-3-sco-10.1177_2050313X251359029 – Supplemental material for Biologic and small molecule therapies for psoriasis in individuals with Down syndrome: Two cases and a systematic review Supplemental material, sj-docx-3-sco-10.1177_2050313X251359029 for Biologic and small molecule therapies for psoriasis in individuals with Down syndrome: Two cases and a systematic review by Michal Moshkovich, Jonah W. Perlmutter and Ronald Vender in SAGE Open Medical Case Reports

sj-docx-4-sco-10.1177_2050313X251359029 – Supplemental material for Biologic and small molecule therapies for psoriasis in individuals with Down syndrome: Two cases and a systematic review Supplemental material, sj-docx-4-sco-10.1177_2050313X251359029 for Biologic and small molecule therapies for psoriasis in individuals with Down syndrome: Two cases and a systematic review by Michal Moshkovich, Jonah W. Perlmutter and Ronald Vender in SAGE Open Medical Case Reports
