# Supplementary material for: Biologic and small molecule therapies for psoriasis in individuals with Down syndrome: Two cases and a systematic review
Source: SAGE Open Med Case Rep. 2025 Jul 22;13:2050313X251359029. doi: 10.1177/2050313X251359029 (PMC12290259; doi:10.1177/2050313X251359029)
Supplement: sj-docx-1-sco-10.1177_2050313X251359029 – Supplemental material for Biologic and small molecule therapies for psoriasis in individuals with Down syndrome: Two cases and a systematic review [file sj-docx-1-sco-10.1177_2050313X251359029.docx]

**Supplementary Table 1.** Search strategy used for literature screening.

Embase Classic+Embase <1947 to 2024 January 7>

Ovid MEDLINE(R) ALL <1946 to 2024 January 7>

| **#** | **Search line** | **Results** |
| --- | --- | --- |
| 1 | (Biologic* or biologic drug* or IL-17 inhibitor* or IL-23 inhibitor* or IL-12 inhibitor* or IL-6 inhibitor* or anti-TNF* or janus kinase inhibitor* or secukinumab or Ixekizumab or etanercept or adalimumab or golimumab or infliximab or ustekinumab or risankizumab or guselkumab or tocilizumab or tofacitinib).mp. [mp=ti, ab, hw, tn, ot, dm, mf, dv, kf, fx, dq, bt, nm, ox, px, rx, ui, sy, ux, mx] | 4,428,601 |
| 2 | (Down syndrome or trisomy 13 or trisomy or chromosomal triplication).mp. [mp=ti, ab, hw, tn, ot, dm, mf, dv, kf, fx, dq, bt, nm, ox, px, rx, ui, sy, ux, mx] | 130,989 |
| 3 | (Psoriasis or psoriatic or pustular psoriasis or plaque psoriasis or inverse psoriasis or guttate psoriasis or nail psoriasis or psoriasiform or psoriatic arthritis or erythrodermic psoriasis).mp. [mp=ti, ab, hw, tn, ot, dm, mf, dv, kf, fx, dq, bt, nm, ox, px, rx, ui, sy, ux, mx] | 214,163 |
| 4 | 1 and 2 and 3 | 64 |
