# Supplementary material for: Biologic and small molecule therapies for psoriasis in individuals with Down syndrome: Two cases and a systematic review
Source: SAGE Open Med Case Rep. 2025 Jul 22;13:2050313X251359029. doi: 10.1177/2050313X251359029 (PMC12290259; doi:10.1177/2050313X251359029)
Supplement: sj-docx-2-sco-10.1177_2050313X251359029 – Supplemental material for Biologic and small molecule therapies for psoriasis in individuals with Down syndrome: Two cases and a systematic review [file sj-docx-2-sco-10.1177_2050313X251359029.docx]

**Identification of studies via databases and registers**

**Studies identified in initial search of OVID EMBASE and MEDLINE Databases** (n=64)

**Identification**

**Duplicate studies removed** (n = 14)

**Screening**

[**Biologic and Small Molecule Treatment for Seborrheic Dermatitis: An Evidence-Based Review**](https://librarysearch.library.utoronto.ca/discovery/fulldisplay?docid=ctx53490185500006196&context=SP&vid=01UTORONTO_INST:UTORONTO&lang=en)

**Studies screened**

(n = 50)

**Studies excluded after title/abstract screening**

(n = 29)

**Studies remaining after title/abstract screening**

(n =21)

**Eligibility**

[**Biologic and Small Molecule Treatment for Seborrheic Dermatitis: An Evidence-Based Review**](https://librarysearch.library.utoronto.ca/discovery/fulldisplay?docid=ctx53490185500006196&context=SP&vid=01UTORONTO_INST:UTORONTO&lang=en)

**Studies excluded after full-text review**

(n =11)

Incorrect Study Design (n = 4)

No Full Text Available (n = 3)

Incorrect Demographics (n = 2)

Incorrect Disease (n = 1)

Non-English Study (n = 1)

**Studies remaining after full text review**

(n =10)

**Included**

The criteria for study inclusion were I) patient(s) with a diagnosis of Down syndrome and any subtype psoriasis II) patient(s) treated with biologics with reported treatment outcomes III) studies that were observational or experimental in nature, including case reports, case series, and retrospective cohort studies IV) studies in the English language

**Supplemental Figure 1.** Flow diagram of literature screening using the Preferred Reporting Items for Systematic Reviews and Meta-Analyses (PRISMA) guidelines. Figure adapted from http://prisma-statement.org.
