# Supplementary material for: Biologic and small molecule therapies for psoriasis in individuals with Down syndrome: Two cases and a systematic review
Source: SAGE Open Med Case Rep. 2025 Jul 22;13:2050313X251359029. doi: 10.1177/2050313X251359029 (PMC12290259; doi:10.1177/2050313X251359029)
Supplement: sj-docx-3-sco-10.1177_2050313X251359029 – Supplemental material for Biologic and small molecule therapies for psoriasis in individuals with Down syndrome: Two cases and a systematic review [file sj-docx-3-sco-10.1177_2050313X251359029.docx]

**Supplementary Table 3.** Treatment outcomes of patients with Down syndrome treated with small molecule or biologic therapies for psoriasis. Abbreviations: CAS, case series; CRS, case report; n, number applicable from total sample; N, total sample size; PASI, psoriasis area severity index.

| **Treatment**  **(%, n/N)** | **Study Design**  **(%, n/N)** | **Outcome (%, n/N)** | **Mean % Change in PASI**  **(n/N)**  ****Weighted average for available date*** | **Therapy Duration, Weeks (n/N)**  ****Weighted average for available date*** |
| --- | --- | --- | --- | --- |
| Adalimumab  (27.0%, 17/63) | CAS (30%, 3/10)  CRS (10%, 1/10) | Recovery  (47.1%, 8/17) | 83.3% (2/8) | 49 (8/17) |
|  |  | No recovery  (52.9 %, 9/17) | 0% (9/9) |  |
| Ustekinumab (20.6%, 13/63) | CAS (20%, 2/10)  CRS (10%, 1/10) | Recovery  (69.2%, 9/13) | 98.5% (7/9) | 72.8 (8/13) |
|  |  | No recovery  (30.8%, 4/13) | 0% (4/4) |  |
| Etanercept (12.7%, 8/63) | CAS (20%, 2/10)  CRS (20%, 2/10) | Recovery  (25%, 2/8) | 83.3% (2/2) | 10 (2/8) |
|  |  | No recovery  (75%, 6/8) | 0% (6/8) |  |
| Secukinumab (9.5%, 6/63) | CAS (20%, 2/10)  CRS (10%, 1/10) | Recovery  (50%, 3/6) | 95.25% (2/3) | 30 (2/5) |
|  |  | No recovery  (50%, 3/6) | 0% (3/3) |  |
| Tofacitinib  (6.3%, 4/63) | CAS (10%, 1/10)  CRS (10%, 1/10) | Recovery (100%, 4/4) | NR | 6 (1/4) |
| Infliximab (4.8%, 3/63) | CAS (10%, 1/10)  CRS (10%, 1/10) | Recovery  (33.3%, 1/3) | NR | 208 (1/3) |
|  |  | No recovery  (66.7%, 2/3) | 0% (2/2) |  |
| Ixekizumab (4.8%, 3/63) | CAS (20%, 2/10) | Recovery  (66.7%, 2/3) | 98.5 % (1/2) | 52 (1/3) |
|  |  | No recovery  (33.3%, 1/3) | 0% (1/1) |  |
| Golimumab (3.2%, 2/63) | CAS (10%, 1/10) | No recovery (100%, 2/2) | NR | NR |
| Guselkumab (4.8%, 3/63) | CAS (20%, 2/10)  CRS (10%, 1/10) | Recovery  (66.7%, 2/3) | 90% (1/3) | 52 (1/3) |
|  |  | No recovery (33.3%, 1/3) | 0% (1/3) |  |
| Risankizumab (3.2%, 2/63) | CAS (20%, 2/10) | Recovery (100%, 2/2) | 98.5% (1/2) | 52 (1/2) |
| Tocilizumab (3.2%, 2/63) | CAS (10%, 1/10) | No recovery (100%, 2/2) | NR | NR |
