# Supplementary material for: Biologic and small molecule therapies for psoriasis in individuals with Down syndrome: Two cases and a systematic review
Source: SAGE Open Med Case Rep. 2025 Jul 22;13:2050313X251359029. doi: 10.1177/2050313X251359029 (PMC12290259; doi:10.1177/2050313X251359029)
Supplement: sj-docx-4-sco-10.1177_2050313X251359029 – Supplemental material for Biologic and small molecule therapies for psoriasis in individuals with Down syndrome: Two cases and a systematic review [file sj-docx-4-sco-10.1177_2050313X251359029.docx]

**Supplemental Table 2.** Cases of patients with Down syndrome diagnosed with psoriasis exposed to biologic therapies. Abbreviations: BID, twice daily; BIW, twice weekly; BSA, body surface area; CAS, case series; CRS, case report; CS, corticosteroids; IV, intravenous; NR, none reported; PO, oral; PASI, Psoriasis Area Severity Index; PR, partial response; q1wk, every 1 week; q2wk, every 2 weeks; q8wk, every 8 weeks; RAD, radiation; SC, subcutaneous; TOP, topical; wk, week

| **Study** | **Study Evidence** | **Sample size** | **Mean age (range)** | **Sex (n)** | **Comorbidities** | **Family History of Psoriasis (n)** | **Age at Diagnosis (Years)** | **Duration of Psoriasis (Months)** | **Nail Involvement (n)** | **Joint Involvement (n)** | **Previous therapies [route] (n)** | **Biologic used [route, dose frequency] (n)** | **Duration of Therapy (weeks)** | **PASI (pre-)/{post-)** | **BSA (pre-)/ (post-)** |
| --- | --- | --- | --- | --- | --- | --- | --- | --- | --- | --- | --- | --- | --- | --- | --- |
| Adamczyk (2016)^3^ | CRS | 1 | 12 | F(1) | NR | NR | 4 | 96 | NR | 1 | Cyclosporine [SC] (1); CS [TOP] (1) | Etanercept [SC, 0.8 mg/kg, BIW](1) | 12 | 41/2 | 78/5.5 |
| Alcaide (2008)^4^ | CRS | 1 | 30 | M(1) | NR | NR | 21 | 108 | 0 | 0 | CS [TOP] (1) | Etanercept [SC, 25 mg, q1wk) | 8 | 14/2 | 30/NR |
| O’Connor (2023)^2^ | CAS | 21 | 24.7 (6-48) | M(13);F(8) | Inflammatory bowel disease (1); ischemic heart disease (1); hepatic steatosis (2); obesity (10) | 5 | NR | NR | 3 | 7 | Phototherapy [RAD] (7); methotrexate [SC] (13); acitretin [PO] (3); cyclosporine [SC] (1); apremilast [PO] (2); fumaric acid esters [TOP] (2) | Etanercept [SC, NR, NR] (5); adalimumab [SC, NR, NR] (11); golimumab [SC, NR, NR] (2); infliximab [IV, NR, NR] (2); ustekinumab [SC, NR, NR] (11); secukinumab [SC, NR, NR] (4); ixekizumab [SC, NR, NR] (2); guselkumab [SC, NR, NR] (2); risankizumab [SC, NR, NR] (1); tocilizumab [SC, NR, NR] (2); tofacitinib [SC, NR, NR] (3) | NR | NR | NR |
| Pham (2021)^5^ | CRS | 1 | 27 | F(1) | Celiac disease (1); hypothydroidism (1) | NR | NR | NR | NR | 1 | NR | Tofacitinib [SC, 5 mg, BID] (1) | 6 | NR/0 | NR/0 |
| Madani (2021)^1^ | CRS | 1 | 33 | M(1) | Asthma (1) | NR | 24 | 108 | NR | 1 | CS [TOP] (1) | Adalimumab [SC, 40 mg, q2wk] (1) | 28 | NR | 10/3 |
| Marmon (2012)^8^ | CAS | 2 | 16 (12-20) | M(2) | Asthma (1); tetralogy of Fallot (1) | 0 | 12 | 48 | NR | NR | Methotrexate [SC] (1) | Etanercept [SC, NR, NR] (1); adalimumab [SC, NR, NR] (2); | NR | NR | 17.5/NR |
| Sugiura (2015)^6^ | CRS | 1 | 15 | M(1) | NR | 1 | 2 | 156 | NR | 1 | Methotrexate [SC](1); CS [TOP] (1); Vitamin D [PO](1) | Infliximab [IV, 3 mg/kg, q8wk] (1); Ustekinumab [SC, NR, NR] (1) | 208 | NR | NR |
| Tancredi (2023)^7^ | CAS | 7 | 22.4 (14-34) | M(5);F(2) | NR | NR | NR | 103.2 | NR | 0 | Phototherapy [RAD] (1); methotrexate [SC] (2); acitretin [PO] (2); cyclosporine [SC] (2) | Adalimumab [SC, NR, NR](3); Ustekinumab [SC, NR, NR](1); Secukinumab [SC, NR, NR] (1); Ixekizumab [IV, NR, NR](1); Risankizumab [SC, NR, NR](1) | 52 | 20/0.3 | 16.6/0.3 |
| Shruthi (2021) | CRS | 1 | 9 | F(1) | Hypothyroidism (1) | NR | 3 | 72 | 0 | 1 | Cyclosporine [SC], CS [TOP] | Secukinumab [SC, 150 mg, q1wk] (1) | 8 | 55/4.4 | 70/NR |
| Ünal (2024) | CRS | 1 | 29 | M(1) | Hypothyroidism (1); Hidradenitis suppurativa (1), Obesity (1) | NR | 13 | 192 | 1 | 1 | Acitretin [PO], CS [TOP], doxycycline [PO], antibiotics [TOP] | Guselkumab [SC, 100 mg, q4wk] (1) | 52 | 10/1 | NR/0 |
